# Probing the Laser Ablation of Black Phosphorus by Raman Spectroscopy

**DOI:** 10.1021/acs.jpcc.1c01443

**Published:** 2021-04-21

**Authors:** Gabriele Faraone, Roberta Sipala, Massimiliano Mariani, Christian Martella, Carlo Grazianetti, Alessandro Molle, Emiliano Bonera

**Affiliations:** † LNESS and Dipartimento di Scienza dei Materiali, Università degli Studi di Milano Bicocca, Via Cozzi-55, I-20125 Milano, Italy; ‡ CNR-IMM, Unità di Agrate Brianza, via C. Olivetti 2, I-20864 Agrate Brianza, Italy

## Abstract

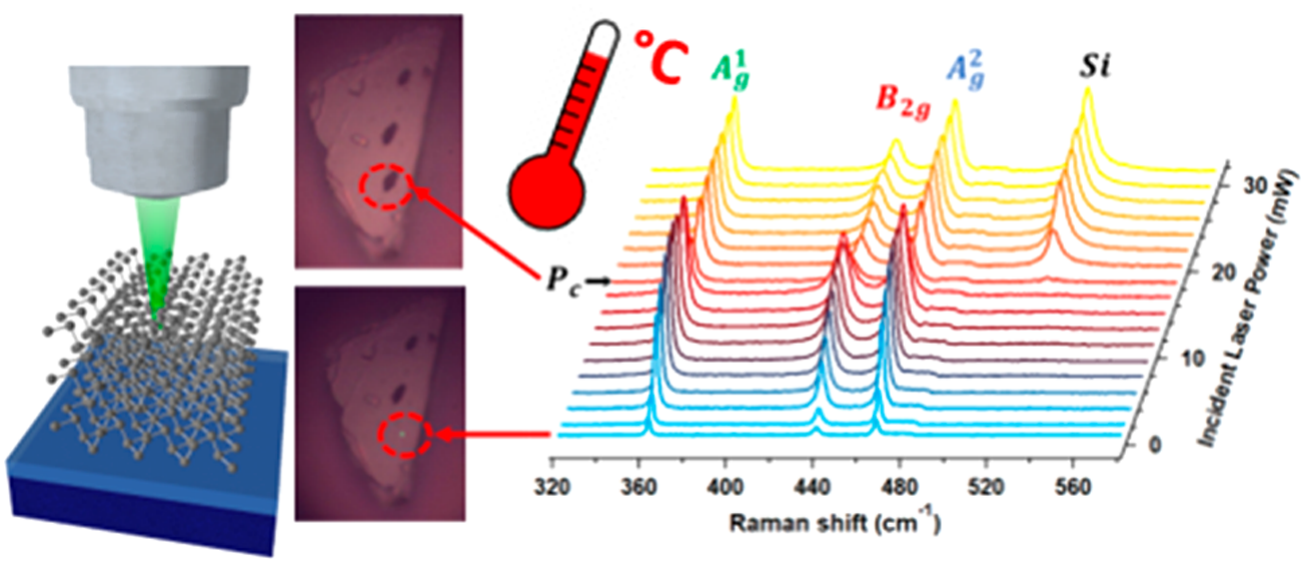

Laser ablation in conjunction with Raman spectroscopy can be used to attain a controllable reduction of the thickness of exfoliated black phosphorus flakes and simultaneous measurement of the local temperature. However, this approach can be affected by several parameters, such as the thickness-dependent heat dissipation. Optical, thermal, and mechanical effects in the flakes and the substrate can influence the laser ablation and may become a source of artifacts on the measurement of the local temperature. In this work, we carry out a systematic investigation of the laser thinning of black phosphorus flakes on SiO_2_/Si substrates. The counterintuitive results from Raman thermometry are analyzed and elucidated with the help of numerical solutions of the problem, laying the groundwork for a controlled thinning process of this material.

## 1 Introduction

Two-dimensional (2D) materials are an emerging research field for a wide range of applications.^1,2^ Among these, orthorhombic black phosphorus (BP) at the 2D limit presents intriguing thickness-dependent optical and electronic properties, such as its direct bandgap ranging from 0.3 eV in the bulk to 1.5 eV in a single layer.^3^ Its strong in-plane anisotropy can also be exploited in innovative device applications.^4−6^ Finally, its high carrier mobility allowed the use of BP in ultrathin field-effect transistors,^7,8^ phototransistors,^9,10^ heterojunctions diodes,^11^ solar cells,^12^ and gas sensors.^13,14^

Bottom-up approaches for the synthesis of 2D phosphorus are limited to the epitaxy of the so-called blue phosphorene allotrope,^15^ which is endowed with an atomically precise thickness but limited by the use of (111)-terminated Au substrates as specific templates. Conversely, thin BP crystals can be produced only by means of top down techniques, such as the mechanical and liquid phase exfoliation,^16−18^ but these methods lack an accurate control of the thickness. Consequently, the exfoliation methods are often complemented with additional thinning steps. Specifically, plasma etching,^19−21^ thermal sublimation,^22^ vacuum,^23^ and rapid thermal annealing^24^ methods have all demonstrated the capability to control the thickness of the flakes down to the single layer.

However, all these thinning approaches do not allow for the control of the thickness on individual flakes, which is necessary when such flakes have an unknown initial thickness. A promising route in this respect is represented by the laser-thinning technique,^25^ demonstrated also for graphite and multilayer MoS_2_.^26−28^ In addition, when coupled with Raman spectroscopy, laser thinning can benefit from the simultaneous control of thickness^29^ and structural properties,^25^ as well as the local measurement of the temperature,^30^ a fundamental parameter for the control of the ablation.

The laser thinning of BP has been investigated in the literature using both high-power ultrafast pulsed laser sources^31,32^ and low-power continuous-wave laser sources.^33,34^ Most of these studies, however, focused on the final results without a quantitative analysis of the optical and thermal effects that influence this process, including parameters such as the thermal coupling with the substrate and the illumination process.

In this work we want to fill this gap by carrying out a Raman thermometric analysis of the laser heating and etching of BP. In particular, we addressed the technologically relevant case of flakes in the range from 1 μm down to 10 nm on SiO_2_/Si substrates. We show that the threshold laser power for the ablation of BP crystals depends on the thickness of the exfoliated flakes and that the conventional experimental determination of the temperature by the Raman peak position is affected by apparent inconsistencies. With a combined experimental and numerical approach, we explain such results by analyzing the role of optical interferences, the effect of the substrate, and the artifacts related to a high-temperature gradient. Finally, we propose that estimating the temperature by the Raman full width at half-maximum (fwhm) can yield more accurate measurements for the thinning temperature of the BP flakes.

## 2 Methods

### 2.1 Experimentals

**Figure 1:**
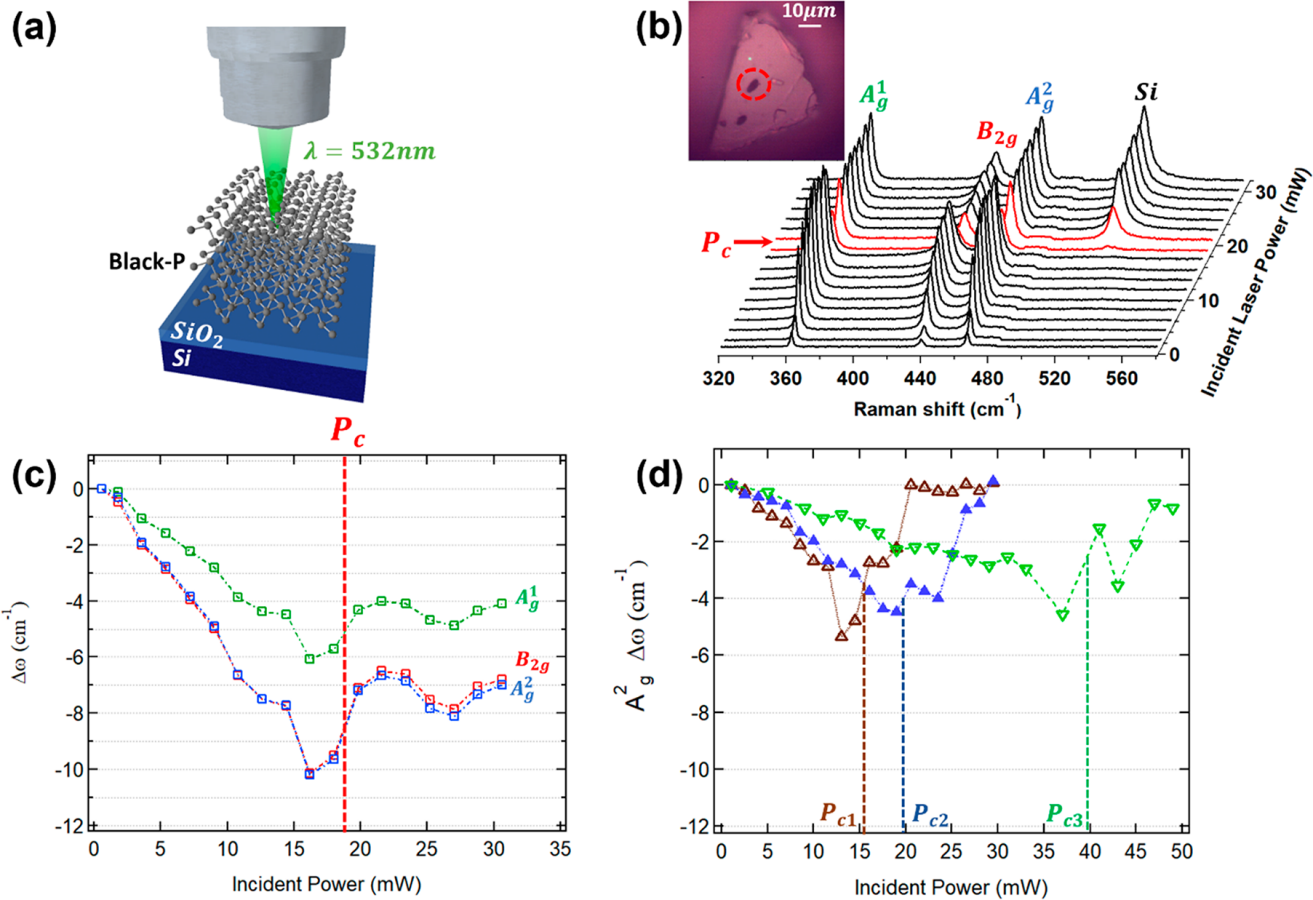
(a) Schematic illustration of a laser-heating experiment carried out on a SiO_2_/Si-supported black phosphorus crystal. (b) Raman spectra acquired during a laser-heating and ablation experiment on a thick flake. Inset: optical image of the flake showing the laser-ablated region (circled in red) (c) Peak position of the A_g_^1^, B_2g_, and A_g_^2^ Raman modes as a function of the incident power from the spectra (b). (d) Raman shift of the A_g_^2^ mode as a function of the incident laser power for three different thin BP flakes. The threshold power for the ablation of the flakes is indicated by *P*_c_, and its position is highlighted by dashed lines in panel (c) and (d).

In order to probe the effects of optical interference, the experiments were carried out on two thickness ranges, thick and thin flakes, as compared to the angle-averaged laser penetration depth in BP, δ_BP_ = 100 nm.^35^ Thick flakes in the range of 0.3–1.2 μm were obtained by liquid phase exfoliation: bulk BP crystals were sonicated for 3 h in an isopropyl alcohol solution, centrifugated for 20 min, and drop-casted on SiO_2_/Si. Thin BP flakes in the range between 10 and 120 nm, instead, were obtained via tape exfoliation and transferred on SiO_2_/Si substrates. The thickness was measured by atomic force microscope (AFM) using a Bruker Dimension Edge instrument in tapping mode equipped with sharp silicon probes (TESPA, Bruker) with a typical radius of curvature in the 8–12 nm range. All the AFM and Raman characterizations were carried out immediately after the exfoliation in order to avoid the degradation of the BP flakes in ambient air^36−38^ (although we did not observe significant differences from fresh and aged samples in the Raman response). Raman spectra were acquired with a Jobin-Yvon T6400 spectrometer with a continuous-wave 532 nm excitation and a 100× (0.9 numerical aperture) objective. The focus diameter was about 0.4 μm. The BP flakes were optically heated as pictured in Figure 1a by controlling the laser incident power with optical filters and a photodiode.

### 2.2 Modeling

Modeling was carried out by solving a stationary heat equation with a finite element method implemented in Mathematica (Wolfram Research). The effects of the substrate were modeled imposing a room-temperature thermal bath boundary condition at the bottom SiO_2_ interface. The anisotropic thermal properties of BP were accounted for by an average in-plane thermal conductivity of 28.8 W m^–1^ K^–1^ and a cross-plane conductivity of 4 W m^–1^ K^–1^.^39−42^

## 3 Results

Figure 1b reports the spectra measured on a thick BP flake. The spectra were collected after an exposure of 20 s for each power. The Raman measurements were performed also varying the exposure time between 1 s and 10 min and keeping the incident power fixed. However, in this situation, we did not observe any significant change in the measured Raman spectra. Thus, a 20 s laser exposure was chosen to provide a common time scale for all the Raman measurements. For low incident powers, *P*_inc_, all the measured spectra present the typical Raman bands of BP: A_g_^1^ (362 cm^–1^), B_2g_ (439 cm^–1^), and A_g_^2^ (467 cm^–1^). However, above the threshold of a critical power *P*_c_ the spectra (highlighted in red in Figure 1b) show a marked reduction of the intensity of the BP bands accompanied by the appearance of the 520 cm^–1^ band of the silicon substrate. This effect is the result of a localized laser ablation of the top layers of the flake similarly to that reported in refs (33 and 34) and can be also observed by a local reflectivity change in the microscope image in the inset of Figure 1b.

In Figure 1c we report the wavenumber variation Δω of the three BP modes, as a function of *P*_inc_. In the low-power region (*P*_inc_ < *P*_c_), the linear red shifts of Δω indicate an increase of the temperature. In this region of the curve the heating process of the samples is reversible, meaning that if we reduce the power, the Raman shift (and the intensity) will reduce accordingly following the same curve. The marked difference observed between the slope, χ_P_ = d(Δω)/d*P*_inc_, of the B_2g_, A_g_^2^, and A_g_^1^ Raman modes can be attributed to the different nature of the corresponding atomic vibrations. The B_2g_ and A_g_^2^ modes are associated with in-plane atomic vibrations along the zigzag and armchair direction of BP, respectively, whereas the A_g_^1^ is mode is associated with the out-of-plane atomic vibrations. Since the behavior of Δω for the three Raman bands is the same (apart from a multiplication constant), we can refer hereafter only to the A_g_^2^ mode, which is the one with the better signal-to-noise ratio and the strongest shifts. The observed threshold power *P*_c_ for the laser ablation of the flake corresponds to an incident power just above the minimum of the Δω curve in agreement with the Raman spectra of Figure 1b. With Δω_c_ we denote the highest |Δω| value measured just before the ablation at *P*_c._. Above *P*_c_, Δω changes in a nonmonotonous way following an oscillating behavior. This apparently puzzling behavior will be explained later in the discussion. For all the thick flakes the initial part of the curve is always the same: *P*_c_ lies in the 16–20 mW range, Δω_c_ is about −10 cm^–1^, and the slope χ_P_ is 6.3 × 10^–2^ cm^–1^/W.

Figure 1d reports that thin BP flakes have a markedly different behavior. As opposed to thick layers, *P*_c_, Δω_c_, as well as the slope χ_P_ show pronounced variations as can be inferred from the Δω of the A_g_^1^ mode measured for three different thin BP layers. In general, as compared to thick layers, *P*_c_ takes higher values (varying above 16 mW), |Δω_c_| is lower, and the slope χ_P_ is lower. Additionally, also in this case Δω changes in a nonmonotonous way above *P*_c_.

**Figure 2:**
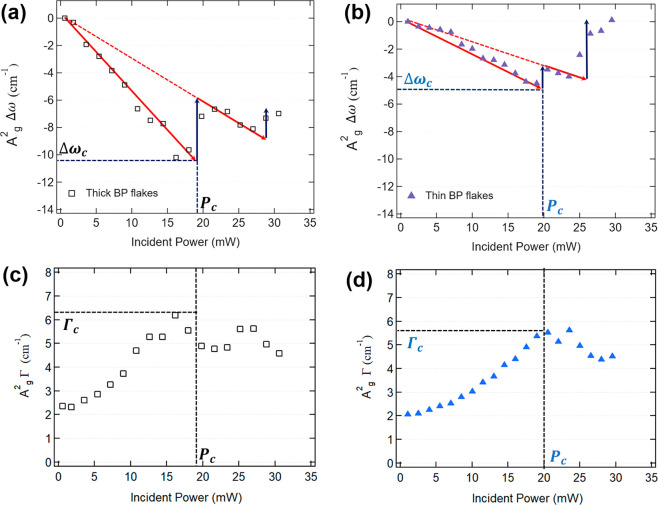
Raman shift and fwhm of the A_g_^2^ mode as a function of the incident laser power for (a)–(c) a thick BP flake and (b)–(d) a thin flake. Blue and red arrows indicate ablation and heating processes, respectively. *P*_c_ indicates the threshold incident laser power.

In Figure 2 we report a direct comparison of the case of a thick and a thin film. The value of Δω can be used to estimate the average local temperature increase Δ*T* caused by the laser optical heating.^43−45^ Using a temperature-shift coefficient χ_T_ = dω/d*T* of 2.76 × 10^–2^ cm^–1^ K^–1^ reported in ref (43), the ablation temperature for thick BP flakes is 410 °C, whereas for the thin BP flake it is 203 °C. Other experimental values reported in the literature for χ_T_^43,46−48^ yield similar results. The typical temperature *T*_c_ reported for the thermal sublimation of BP lies within the 350–400 °C range.^22,49,50^ This is compatible with the heating of the thick BP film but not with the heating of the thin BP film, suggesting that for the latter some other effects must be accounted for.

Figure 2c and d show the power dependence of Γ, namely, the fwhm of the A_g_^2^ band. The values reported correspond to panels (a) and (b), respectively. We notice again that in the low-power region Γ increases monotonically. Since the fwhm is a monotonic increasing function of the temperature,^43,45,51^ the behavior of Γ corroborates the temperature increase within the Raman-probed region also observed from the Raman shifts at *P*_inc_ < *P*_c_. However, in this case the value of Γ_c_, the highest Γ value measured just before the ablation at *P*_c_, is similar for the thick and thin BP films (6.3 ± 0.1 and 5.6 ± 0.1 cm^–1^, respectively). This finding suggests that the ablation temperature derived from Γ is indeed the same for thick and thin flakes. This is reasonable since we expect that the sublimation temperature of BP should not depend sensitively on the flake thickness in the multilayer regime. However, this fact still does not help to explain the anomalous behavior observed for Δω_c_. A detailed understanding of this behavior involves a more in-depth discussion of the process of absorption and diffusion of the incident optical power, as well as a modeling of the process of Raman measurement (see the next section).

Once we observe that for thinner samples Δω_c_ is a function of thickness, the apparently incoherent behavior consisting of backward and forward shifts of Δω observed for *P*_inc_ > *P*_c_ can be explained as follows. The samples are heated until the local temperature is above *T*_c_. Then, there is an ablation of material that locally reduces the thickness of the BP flake. Each time that BP is thinned down, the material is characterized by a different χ_P_ and a lower Δω_c_. With this new thickness, the power must increase further in order to reach again the critical temperature. The heating of the thinned flake continues until we observe the occurrence of a second ablation sequence, with a further change in χ_P_ and Δω_c_. In Figure 2 such cycles are highlighted by an alternation of red (heating) and blue (ablation) arrows. For *P*_inc_ > *P*_c_, the corresponding behavior of Γ reproduces the sequence observed on the Δω curves. However, the modulation in the Γ curves for *P*_inc_ > *P*_c_ is less pronounced than the modulation observed in the Δω curves. Additionally, we notice that the value of Γ_c_ remains nearly the same after each ablation sequence of the flake. This fact again shows that Γ is a better parameter to provide a local probe of the thinning temperature of BP.

## 4 Discussion

### 4.1 Heat and Temperature Distribution

**Figure 3:**
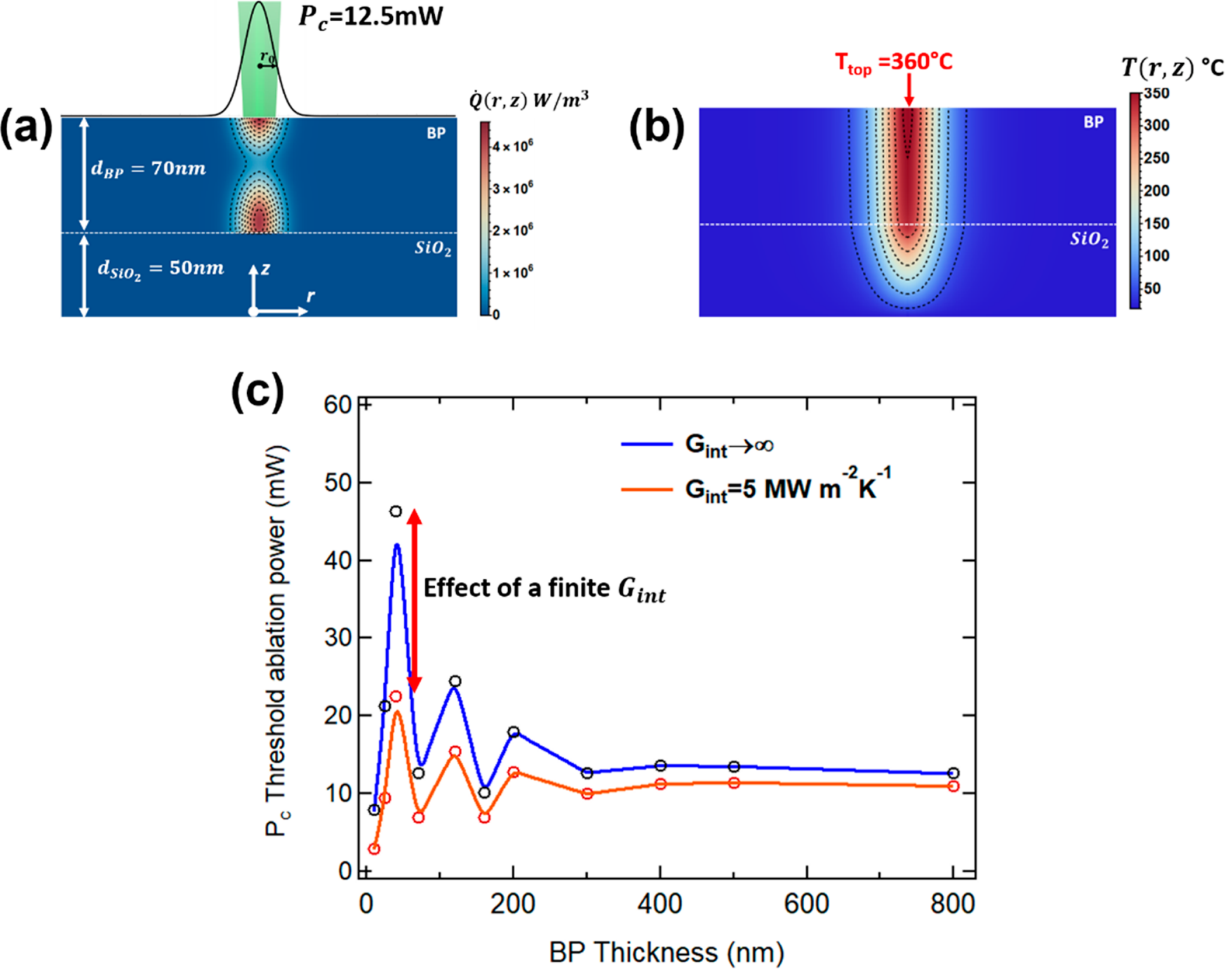
(a) Local heat distribution and (b) temperature for a 70 nm BP flake supported on a SiO_2_ (50 nm)/Si substrate. The incident power of 12.5 mW induces a 360 °C temperature on the top, corresponding to the ablation temperature. (c) Plot of the threshold ablation power *P*_c_ as a function of the black phosphorus thickness. The blue and red lines show the case of an ideal and realistic thermal coupling at the BP/SiO_2_ interface, respectively.

We numerically calculated the temperature generated in the BP flakes by the optical absorption of the laser. For this purpose, we modeled the inhomogeneous heat generated by the laser absorption inside the material by a position-dependent heat source term *Q̇*(*r*, *z*):^52^
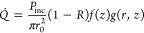
1where *g*(*r*, *z*) is the Gaussian laser beam intensity profile, *r*_0_ is the laser beam radius, and *R* is the reflectance calculated on the BP surface. The function *f*(*z*) describes both the attenuation due to absorption and the modulation due to multiple interference among the rays reflected at the interfaces (see ref (52)). Figure 3a reports the plot of the heat source term *Q̇* in the case of a 70 nm thick BP flake. Inside the BP region, *Q̇* is attenuated exponentially in the *z*-direction and modulated by oscillations caused by multiple interferences. Inside the SiO_2_ region, instead, the heat-source term is always zero since SiO_2_ is optically transparent to the incoming radiation, and therefore, no heat can be generated in its volume. For thick flakes the interference is negligible, and *f*(*z*) follows the Beer–Lambert attenuation law.

Figure 3b plots the temperature corresponding to the heat source in Figure 3a. The incident optical power *P*_inc_ was chosen to induce a surface temperature at the threshold of the sublimation of BP. Within the range of temperatures reported in the literature, we arbitrarily chose the value of 360 °C. Therefore, this value of *P*_inc_ corresponds to the *P*_c_ value observed in the laser-thinning experiments.

### 4.2 Threshold Ablation Power

The blue curve of Figure 3c shows the values of *P*_c_ as a function of the flake thickness. The threshold ablation power is characterized by an oscillating behavior as a function of the thickness, especially in the case of thin flakes. This oscillating behavior of *P*_c_ is a consequence of the interferential modulation of the absorbed light and explains the differences in the threshold ablation power observed on thick and thin BP flakes. The modulation stops around 300 nm, which is consistent with our findings on thick flakes.

While the blue curve is calculated with an infinite thermal boundary conductance, the orange curve refers to a calculation performed including the effects of a finite value for the thermal boundary conductance *G*_int_ = 5 MW m^–2^ K^–1^ at the BP/SiO_2_ interface. This value, although very low,^53^ has the same order of magnitude of the thermal boundary conductance experimentally measured at the interfaces between highly dissimilar materials.^54^ Although the qualitative behavior is the same, we see that a low thermal boundary conductance introduces a change in the modulation of *P*_c_ that turns out to be more pronounced in thin rather than thick BP flakes. This fact indicates that for thin BP flakes the heat dissipation mechanism is governed also by the thermal coupling with the SiO_2_ interface rather than only by the intrinsic thermal properties of BP.

### 4.3 Analysis of the Δω Trend

**Figure 4:**
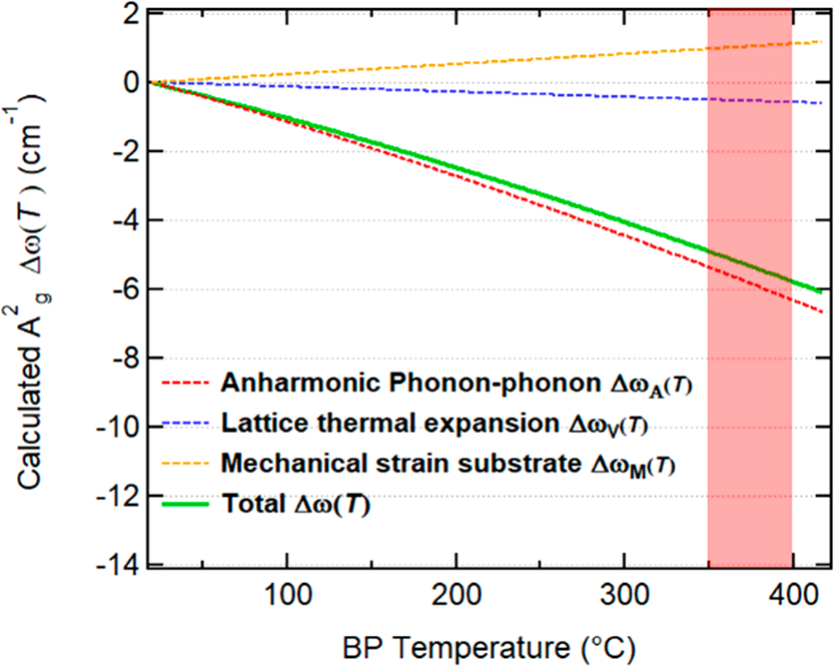
Calculated temperature dependence of the Raman shift of the A_g_^2^ mode obtained as the sum of the phonon–phonon anharmonic decay, lattice thermal expansion, and mechanical strain of the substrate contributions. Sublimation occurs in the range of temperatures marked by the red band.

The temperature dependence of the BP Raman peaks is generally described as the result of three effects:^51,55^
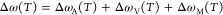
2Δω_A_(*T*) is the shift caused by the decay of phonons because of the phonon–phonon anharmonic interaction.^45,51^ The second term Δω_V_(*T*) is related to the BP lattice thermal expansion equation.^51^ Δω_M_(*T*) describes the effect of the mechanical strain caused by the thermal expansion coefficient mismatch between the BP flake and the underlying SiO_2_/Si substrate.^55^ The second and third contributions of eq 2 may introduce a thickness dependence in the Δω because typically the mechanical properties of thinner BP films are more influenced by the adjacent substrate. The evaluation of the three terms can be observed in Figure 4 in the 20–400 °C temperature range, which was calculated on the basis of the data reported in the literature.^43,51^ At *T*_c_, Δω_A_(*T*) brings the major contribution of about 6 cm^–1^. The mechanical strain effect, reflected in Δω_M_(*T*), contributes with a positive shift of about 1 cm^–1^. The relatively less important contribution of Δω_V_(*T*) instead is less than 1 cm^–1^.

**Figure 5:**
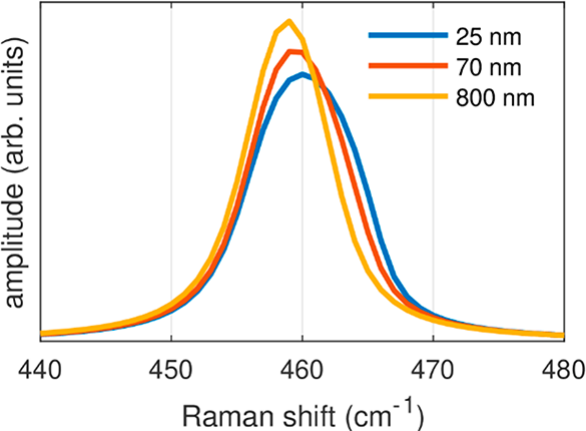
Simulated spectral line shapes of the A_g_^2^ Raman mode assuming a temperature of 360 °C at the focal point on the top surface of three black phosphorus flakes with different thicknesses. The blue shift of the Raman frequency toward high wavenumbers in thinner flakes is due to the high-temperature gradient.

An additional cause that may influence the value of Δω is related to the existence of high-temperature gradients in thin BP films caused by the heat sink effect of the supporting substrate. Each Raman spectrum is the result of the scattering from different regions of the sample illuminated by the laser. When the temperature gradient is high, each region inside the scattering volume of the sample contributes a significantly different Δω. Figure 5 reports the A_g_^2^ Raman line shapes simulated for three BP flakes with different thicknesses. Each of the three spectra is obtained by summing up all the contributions from the regions inside the BP flake corresponding to the finite elements of the simulation.^56,57^ In the sum, each contribution has been weighted with a position-dependent factor *f*(*z*)^2^*g*(*r*, *z*) that accounts for the nonuniform collection of the Raman scattered radiation inside the illuminated region of the flake. Finally, the calculations are performed assuming a surface temperature of 360 °C for the three BP flakes. The sum of spectra from hot and cold regions in the flake yields an apparent blue shift of the overall Raman peak, which is up to 1 cm^–1^ for the thinnest films. This is a pure artifact of the averaging process that occurs during the collection of the Raman-scattered light from the sample.

### 4.4 Analysis of Γ

Unlike Δω, the temperature dependence of Γ is related only to the decay of optical phonons due to the phonon–phonon anharmonic interaction.^43,45,51^ Γ is not affected by the volumetric expansion of the BP lattice and by the mechanical strain effect. Using the parametric expression of Γ(T) reported in ref (51) for a 20 nm thick BP flake, we can extract the ablation temperature of BP using the highest value of Γ measured just before the ablation, Γ_c_, obtaining *T*_c_ = 360 °C for the thin BP flake and *T*_c_ = 420 °C for the thick BP flake in Figure 2. Both these values agree well with the thermal sublimation temperature of BP.^22,49,50^ This observation resolves the contradiction in the ablation temperatures probed using the Δω_*c*_ values in thick and thin BP flakes. For *P*_inc_ > *P*_c_ the ablation process reduces the thickness of the flakes to values for which the absorption of the radiation is lower due to the combined effect of optical interferences and heat sink of the supporting Si/SiO_2_ substrate. The measured temperature in the flake is, therefore, lower than the value determined at the incident power where |Δω| and Γ assume the highest value. This change of the temperature can therefore be observed by both a slight decrease in the Γ values and the corresponding blue shift of Δω at *P*_inc_ > *P*_c._ However, our results show that despite the fact that Δω is a quantity that can be measured with a better accuracy it can be considered as a reproducible parameter for the local measurement of temperature for thick BP flakes only. In the case of thin flakes, instead a more trustworthy measurement of the temperature is given by the value of Γ.

## 5 Conclusions

Laser ablation in conjunction with local measurement of the temperature by Raman spectroscopy can be used as a method for the controlled thinning of BP. We carried out a systematic study on multilayer BP flakes supported on SiO_2_/Si substrates aiming to observe the variation of the Raman spectra as a function of the increasing laser power and the advancement of the ablation process.

We found that the threshold incident optical power for the laser ablation is reproducible for all thick layers but not for flakes below hundreds of nanometers. In this thickness regime the threshold laser ablation power is an oscillating function of the thickness since it is affected by the interplay between the thermal coupling and optical interference effects with the supporting substrate.

Additionally, we proved that a simple model for the temperature dependence of the Raman shifts may lead to discrepancies when used to probe the temperatures in multilayer BP flakes, especially at high incident optical powers close to the ablation threshold. In particular, beyond the contributions due to the phonon–phonon anharmonic coupling, also the thermal expansion of the BP lattice and the mechanical strain effect with the supporting substrate play a role. Moreover, we also observed that the heat-sink effect of the substrate together with the nonuniform Raman collection efficiency from the flakes may alter the Raman shift values and the predicted temperatures at the ablation.

We considered also the temperature versus power dependence of the fwhm of the Raman bands. This quantity is mostly sensitive to the anharmonic effects of the temperature and can be used as a reliable indicator for its measurement at all the thickness ranges. In this respect, the present study not only provides a better understanding of the laser-thinning process in BP but also envisages a correct methodology for the correct determination of the temperature from Raman optical thermometry experiments.
